# Increased risk of atrial fibrillation in young adults with gout: a nationwide cohort study

**DOI:** 10.3389/fcvm.2026.1862887

**Published:** 2026-07-07

**Authors:** Eunsong Kang, Kyung-Do Han, Gwan Gyu Song, Jae Hyun Jung, Youngho Lee

**Affiliations:** 1 Department of Rheumatology, Korea University Ansan Hospital, Ansan, Republic of Korea; 2 Department of Statistics, Soongsil University, Seoul, Republic of Korea; 3 Department of Rheumatology, Korea University Guro Hospital, Seoul, Republic of Korea; 4 Department of Rheumatology, Korea University Anam Hospital, Seoul, Republic of Korea

**Keywords:** atrial fibrillation, cardiovascular risk, gout, nationwide cohort study, young adults

## Abstract

**Background:**

Gout is a systemic inflammatory disorder increasingly associated with cardiovascular comorbidities. However, its potential relationship with atrial fibrillation (AF), especially in young adults, has not been fully delineated. To investigate the association between gout and incident AF in a large nationwide cohort of Korean adults aged 20–39 years.

**Methods:**

This retrospective cohort study analyzed 6,506,721 individuals from the Korean National Health Insurance Service (NHIS) database. Gout and AF were identified using ICD-10 codes M10 and I48, respectively. Cox proportional hazards regression models were used to estimate hazard ratios (HRs) and 95% confidence intervals (CIs) for incident AF after adjusting for demographic, lifestyle, and clinical factors.

**Results:**

During a median follow-up of 12.59 years, gout was associated with a significantly increased risk of AF (fully adjusted HR, 1.44; 95% CI, 1.31–1.58). The cumulative incidence of AF was significantly higher in the gout group (log-rank *P* < .001). Subgroup analyses demonstrated stronger associations among non-obese individuals and those with chronic kidney disease (CKD).

**Conclusions:**

Gout was associated with an increased risk of atrial fibrillation among young adults. These findings suggest that gout may serve as a clinically relevant risk marker for AF, underscoring the need for proactive cardiovascular risk assessment in this population.

## Background

Gout is a chronic inflammatory disease caused by the deposition of monosodium urate crystals in joints and tissues, resulting from longstanding hyperuricemia. Although classically recognized for causing intermittent attacks of severe joint pain and inflammation, gout is now increasingly understood as a systemic disease with important extra-articular implications—particularly in the cardiovascular system (1–4).

Hyperuricemia and gout have been associated with an increased risk of various cardiovascular conditions, including hypertension, coronary artery disease, and heart failure. These associations are thought to arise from multiple pathogenic mechanisms. Uric acid may promote endothelial dysfunction, oxidative stress, and systemic inflammation through activation of the NLRP3 inflammasome and production of reactive oxygen species (ROS) (5–7). These factors contribute to electrical and structural remodeling of the atria, thus increasing vulnerability to atrial fibrillation (AF). Moreover, hyperuricemia may directly impair intracellular ion homeostasis and prolong action potentials, further promoting arrhythmogenesis.

Despite these plausible mechanisms, the association between gout and atrial fibrillation (AF) has not been extensively studied in younger populations. AF, a common arrhythmia associated with significant morbidity and mortality, is predominantly considered a disease of older adults (8–11); however, its prevalence is rising among younger individuals, likely due to increased detection and the growing prevalence of contributing factors such as obesity, metabolic syndrome, and sedentary lifestyles. When AF develops at a younger age, the long-term burden can be substantial due to prolonged exposure to arrhythmia-related complications over the life course. Although younger adults are less likely to harbor traditional structural cardiac abnormalities, AF in this population may instead reflect the influence of systemic inflammatory or metabolic conditions—such as gout (12–14). Notably, early-onset AF has been associated with adverse outcomes including heart failure and stroke, especially in the presence of coexisting risk factors. Therefore, identifying non-traditional contributors to AF in younger adults is essential for effective prevention and risk stratification.

In this context, we aimed to assess whether gout is associated with an increased risk of incident AF in adults aged 20–39 years using a comprehensive, nationwide health insurance database in Korea. Furthermore, we explored whether this association is modified by demographic or clinical characteristics such as sex, obesity, and chronic kidney disease (CKD), given their known roles in both AF and gout pathophysiology.

## Methods

### Study design and data source

This retrospective cohort study used data from the Korean National Health Insurance Service (NHIS) database, which provides universal coverage to over 97% of Koreans. The NHIS includes sociodemographic characteristics, inpatient and outpatient records, ICD-10 diagnostic codes, procedures, prescription claims, and health screening results containing self-reported questionnaires, anthropometric measurements, and laboratory data. This structure allows comprehensive, longitudinal tracking of health outcomes nationwide.

We included individuals aged 20–39 years who underwent general health screening between January 1, 2009, and December 31, 2012. The first screening date during this period was the index date. To reduce reverse causality, we excluded those with any atrial fibrillation (AF) diagnosis before the index date and those missing key variables (smoking, alcohol, physical activity, BMI, or laboratory data). The final cohort comprised 6,506,721 young adults (Supplementary Figure S1).

This study was approved by the Institutional Review Board of Kangbuk Samsung Hospital (IRB No. 2025-04-033) and followed the Declaration of Helsinki and the STROBE (Strengthening the Reporting of Observational Studies in Epidemiology) guidelines.

### Exposure and outcome definitions

Gout was defined using ICD-10 code M10 in inpatient or outpatient claims. Participants were classified as having gout if they had ≥2 outpatient visits or ≥1 hospitalization with this code within 3 years before the index date. This definition has been validated in Korean NHIS data with positive predictive values above 85%. Individuals without any M10 code during the same period comprised the non-gout group.

The primary outcome was incident AF, defined using ICD-10 code I48 (including paroxysmal, persistent, and chronic AF). AF required ≥2 outpatient or ≥1 hospitalization claims after the index date. This case definition has demonstrated high specificity and acceptable sensitivity in Korean administrative data. Participants were followed from the index date until AF, death, or December 31, 2023. To minimize reverse causality, individuals diagnosed with AF within one year of the index date were excluded.

### Covariates

We included a broad range of covariates potentially associated with both gout and AF. Demographic variables included age, sex, household income (quartiles), smoking status (never, former, current), alcohol consumption (none, mild <30 g/day, heavy ≥30 g/day), and regular physical activity (≥3 moderate-to-vigorous sessions per week). Clinical variables included BMI (<25 or ≥25 kg/m^2^, Asia-Pacific criteria), and comorbidities such as hypertension, diabetes, dyslipidemia, and chronic kidney disease (CKD). Hypertension was defined as systolic BP ≥140 mmHg, diastolic BP ≥90 mmHg, or antihypertensive medication use; diabetes as fasting glucose ≥126 mg/dL or antidiabetic medication use; dyslipidemia as total cholesterol ≥240 mg/dL or lipid-lowering therapy; and CKD as estimated glomerular filtration rate (eGFR) <60 mL/min/1.73m^2^, calculated using the Modification of Diet in Renal Disease (MDRD) equation.

Laboratory values including fasting glucose, total cholesterol, and eGFR were extracted from the NHIS health screening database for descriptive analysis but were not included in multivariable models due to potential collinearity with the diagnosis-based definitions of comorbidities.

### Statistical analysis

Baseline characteristics of participants were summarized by gout status using means and standard deviations for continuous variables and proportions for categorical variables. Incidence rates of AF were calculated per 1,000 person-years. Cox proportional hazards regression models were used to estimate hazard ratios (HRs) and 95% confidence intervals (CIs) for the association between gout and incident AF. We constructed three hierarchical models: Model 1 (unadjusted), Model 2 (adjusted for age and sex), and Model 3 (fully adjusted for all demographic, lifestyle, and clinical covariates).

The proportional hazards assumption was verified using Schoenfeld residuals. Multicollinearity between covariates was assessed using the variance inflation factor (VIF), and no significant collinearity was found. Subgroup analyses were conducted to examine effect modification by sex, obesity (BMI ≥25 kg/m^2^), hypertension, diabetes, dyslipidemia, smoking status, alcohol consumption, physical activity, and CKD. For each subgroup, interaction terms were tested to assess statistical significance of heterogeneity.

We also conducted sensitivity analyses excluding individuals with follow-up <2 years and stratified analyses by age strata (20–29 vs. 30–39 years). All analyses were performed using R version 4.2.1 (R Foundation for Statistical Computing, Vienna, Austria) and SAS Enterprise Guide version 7.1 (SAS Institute, Cary, NC, USA). A two-tailed *p*-value of <0.05 was considered statistically significant.

## Results

A total of 6,506,721 individuals aged 20 to 39 years were included in the final analysis. Among these, 35,742 (0.55%) had a diagnosis of gout prior to the index date. Baseline characteristics of the study population stratified by gout status are presented in Table 1. Compared with the non-gout group, individuals with gout were more likely to be male (88.83% vs. 59.29%) and had a higher mean age (33.37 vs. 30.84 years). The gout group also exhibited a higher prevalence of traditional cardiovascular risk factors, including current smoking (48.57% vs. 34.76%), obesity (BMI ≥25 kg/m^2^: 56.85% vs. 26.26%), hypertension (24.70% vs. 7.37%), diabetes mellitus (5.50% vs. 1.91%), dyslipidemia (20.62% vs. 6.75%), and chronic kidney disease (4.35% vs. 2.29%) (all *P* < 0.001). Laboratory data reflected similar trends, with higher fasting glucose and total cholesterol levels, and lower eGFR values in the gout group.

**Table 1:** Baseline characteristics of the study population by gout Status.

Characteristics	Total (*n* = 6,506,721)	Non gout (*n* = 6,470,979)	Gout (*n* = 35,742)	*P*-value
Age, years				<.0001
20–29	2741511 (42.13)	2734054 (42.25)	7,457 (20.86)	
30–39	3765210 (57.87)	3736925 (57.75)	28,285 (79.14)	
Sex, Male	3868519 (59.45)	3836768 (59.29)	31,751 (88.83)	<.0001
Smoking				<.0001
Never	3567411 (54.83)	3555171 (54.94)	12,240 (34.25)	
Ex smoker	672891 (10.34)	666750 (10.3)	6,141 (17.18)	
Current smoker	2266419 (34.83)	2249058 (34.76)	17,361 (48.57)	
Alcohol consumption				<.0001
Non	2455032 (37.73)	2444109 (37.77)	10,923 (30.56)	
Mild	3478670 (53.46)	3459058 (53.45)	19,612 (54.87)	
Heavy	573019 (8.81)	567812 (8.77)	5,207 (14.57)	
Regular Exercise	835457 (12.84)	829661 (12.82)	5,796 (16.22)	<.0001
BMI_Level				<.0001
<18.5	493161 (7.58)	492276 (7.61)	885 (2.48)	
<23	3042403 (46.76)	3034614 (46.9)	7,789 (21.79)	
<25	1251196 (19.23)	1244449 (19.23)	6,747 (18.88)	
<30	1444998 (22.21)	1430232 (22.1)	14,766 (41.31)	
≥30	274963 (4.23)	269408 (4.16)	5,555 (15.54)	
Low Income	1384456 (21.28)	1378968 (21.31)	5,488 (15.35)	<.0001
Diabetes mellitus	125511 (1.93)	123544 (1.91)	1,967 (5.5)	<.0001
Hypertension	486038 (7.47)	477208 (7.37)	8,830 (24.7)	<.0001
Dyslipidemia	444027 (6.82)	436657 (6.75)	7,370 (20.62)	<.0001
CKD	149590 (2.3)	148037 (2.29)	1,553 (4.35)	<.0001
Age, years	30.86 ± 4.99	30.84 ± 4.99	33.37 ± 4.34	<.0001
BMI, kg/m2	23 ± 3.61	22.98 ± 3.6	25.87 ± 4.17	<.0001
Waist Circumference, cm	77.51 ± 10	77.46 ± 9.98	85.77 ± 10.59	<.0001
Fasting Glucose, mg/dL	90.87 ± 16.62	90.85 ± 16.57	95.65 ± 23.1	<.0001
Systolic BP, mmHg	117.72 ± 13.18	117.68 ± 13.16	124.83 ± 14.77	<.0001
Diastolic BP, mmHg	73.77 ± 9.45	73.74 ± 9.43	78.82 ± 10.83	<.0001
Total Cholesterol, mg/dL	184.56 ± 33.81	184.48 ± 33.77	198.3 ± 39.02	<.0001
HDL Cholesterol, mg/dL	57.31 ± 21.95	57.34 ± 21.96	51.53 ± 18.98	<.0001
LDL Cholesterol, mg/dL	104.69 ± 34.48	104.66 ± 34.46	110.44 ± 37.14	<.0001
GFR, mL/min/1.73m2	101.75 ± 21.63	101.79 ± 21.62	95.87 ± 23.03	<.0001
*Triglyceride	96.75 (96.71–96.8)	96.51 (96.47–96.56)	151.84 (150.83–152.86)	<.0001

Baseline demographic, lifestyle, and clinical characteristics of the study population, stratified by presence or absence of gout. Values are presented as number (%) for categorical variables and mean ± standard deviation for continuous variables unless otherwise noted.

*P*-values were derived from chi-square test for categorical variables and *t*-test (or geometric mean comparison) for continuous variables.

*Triglyceride is presented as geometric mean (95% confidence interval).

During a median follow-up of 12.59 years (interquartile range [IQR]: 11.49–13.23 years), a total of 446 incident cases of atrial fibrillation (AF) occurred in the gout group, while 35,227 cases were observed in the non-gout group. The incidence rate of AF was markedly higher in the gout group at 1.03 per 1,000 person-years, compared to 0.44 per 1,000 person-years in the non-gout group. The corresponding incidence rate ratio (IRR) was 2.34 (95% CI: 2.10–2.59), consistent with the crude hazard ratio.

In the initial analysis without covariate adjustment, gout was significantly associated with an increased risk of AF, with a hazard ratio (HR) of 2.324 (95% CI: 2.117–2.552; *p* < 0.0001). Adjustment for age and sex attenuated the estimate but the association remained robust (HR 1.731; 95% CI: 1.577–1.901; *p* < 0.0001). Further adjustment for behavioral factors (smoking status, alcohol consumption, physical activity), BMI, and comorbidities (hypertension, diabetes, dyslipidemia, and CKD) yielded a fully adjusted HR of 1.438 (95% CI: 1.310–1.580; *p* < 0.0001), confirming the independent association between gout and increased AF risk (Table 2).

**Table 2:** Incidence and risk of atrial fibrillation in individuals With and without gout.

Group	*N*	Events	Person-years	Incidence rate (per 1,000 person-years)	Model 1 HR (95% CI)	Model 2 HR (95% CI)	Model 3 HR (95% CI)
Non gout	6,470,979	35,227	79,552,452	0.44	1 (Ref.)	1 (Ref.)	1 (Ref.)
Gout	35,742	446	434,995	1.03	2.324 (2.117, 2.552)	1.731 (1.577, 1.901)	1.438 (1.310, 1.580)
*P*-value					<.0001	<.0001	<.0001

Incidence rates are presented per 1,000 person-years. Hazard ratios (HRs) were calculated using Cox proportional hazards regression models.

Model 1: Unadjusted.

Model 2: Adjusted for age and sex.

Model 3 (Fully adjusted model): Adjusted for age, sex, body mass index (BMI), income level, smoking status, alcohol consumption, regular physical activity, diabetes mellitus, hypertension, dyslipidemia, and chronic kidney disease (CKD).

**Figure 1:**
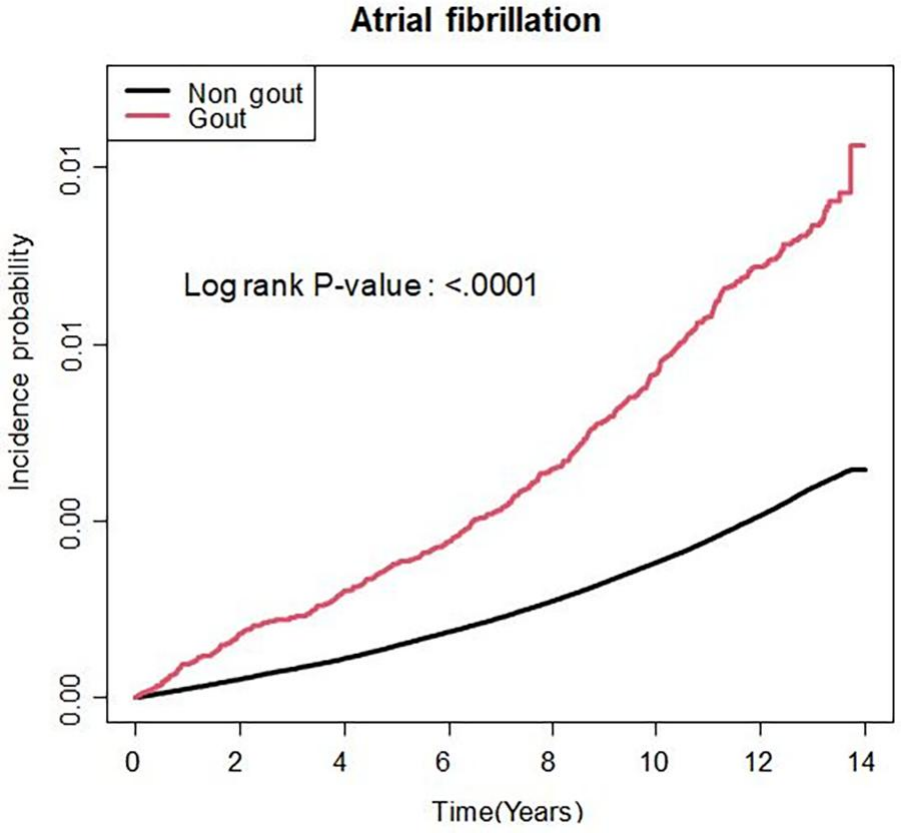
Cumulative incidence of atrial fibrillation in individuals with and without gout. Kaplan–Meier curves comparing the cumulative incidence of atrial fibrillation (AF) between individuals with gout and those without gout. Gout was associated with a significantly higher incidence of AF over time (log-rank *P*-value < .0001).

Kaplan–Meier survival analysis demonstrated a significantly higher cumulative incidence of AF in the gout group throughout the follow-up period (Figure 1; log-rank *p* < 0.0001). The survival curves began to diverge within the first few years and continued to separate progressively over time. At 5 years of follow-up, the cumulative incidence of AF was 0.36% in the gout group vs. 0.15% in the non-gout group. By 10 years, this difference widened further to 0.72% vs. 0.31%, respectively. The log-rank test confirmed that these differences were statistically significant (*p* < 0.0001).

**Figure 2:**
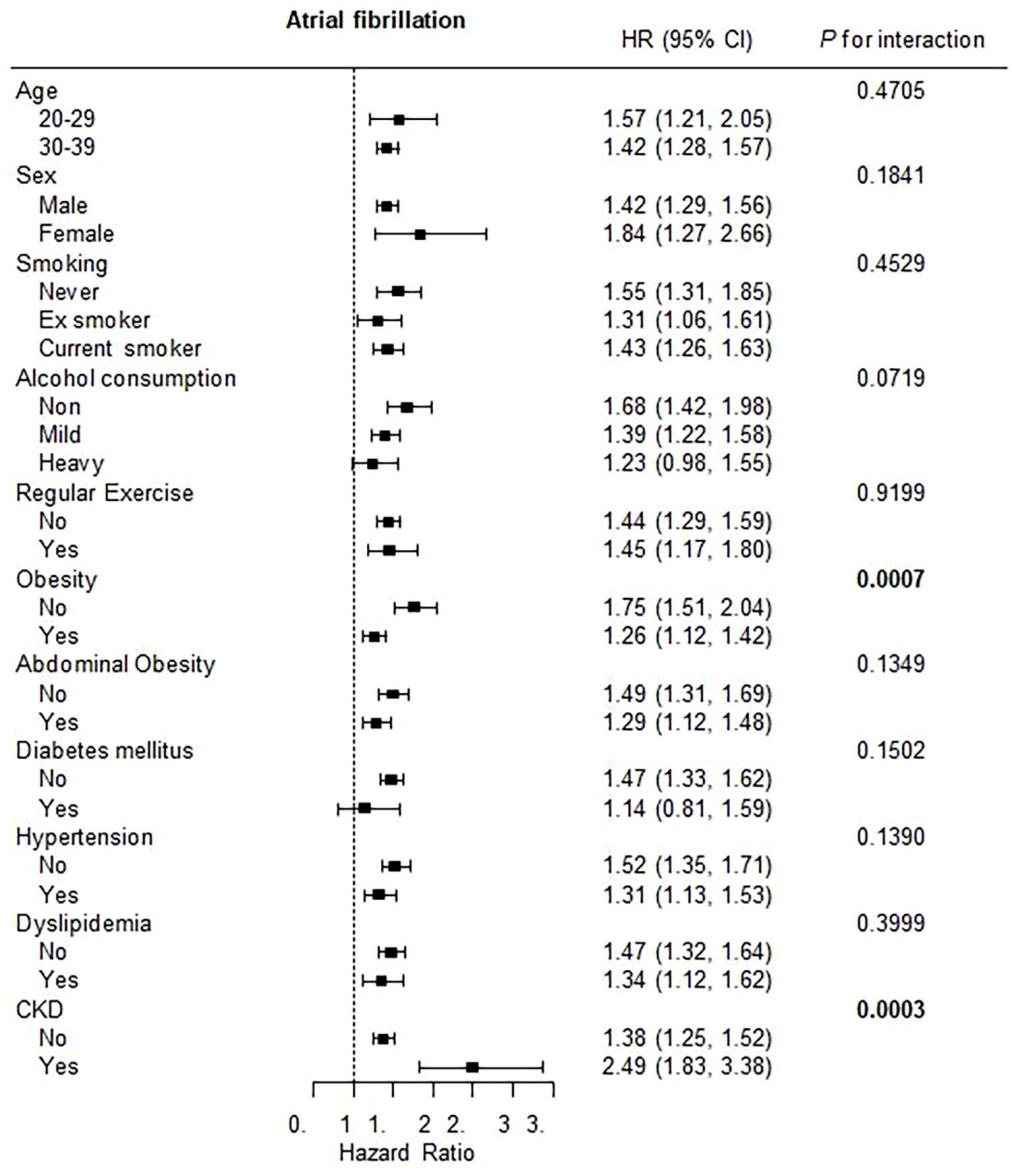
Subgroup analysis of the association between gout and incident atrial fibrillation. Forest plot of adjusted hazard ratios (HRs) with 95% confidence intervals for incident atrial fibrillation across predefined subgroups. All HRs are derived from multivariable Cox proportional hazards models adjusted for age, sex, BMI, income, smoking status, alcohol consumption, regular exercise, diabetes mellitus, hypertension, dyslipidemia, and chronic kidney disease (Model 3). *P*-values for interaction test whether the association between gout and AF significantly differed across subgroup strata.

Subgroup analyses were conducted to examine whether the association between gout and AF varied according to demographic or clinical characteristics (Figure 2). Across most subgroups, including age strata, sex, smoking status, alcohol consumption, physical activity, and the presence of hypertension, diabetes, or dyslipidemia, the association between gout and AF was generally consistent, with no significant interaction effects detected.

However, two clinically significant effect modifiers of the association were identified. First, obesity significantly modified the relationship between gout and AF. Among non-obese individuals (BMI <25 kg/m^2^), gout was associated with a 75% increased risk of AF (HR 1.75; 95% CI: 1.51–2.04), whereas among obese individuals (BMI ≥25 kg/m^2^), the risk was lower (HR 1.26; 95% CI: 1.12–1.42), with a statistically significant interaction (*p* for interaction = 0.0007). This pattern indicates that the pro-inflammatory milieu of gout exerts a greater arrhythmogenic influence in the absence of competing metabolic risk factors such as obesity.

Second, CKD appeared to amplify the arrhythmic risk associated with gout. In individuals with CKD, the risk of AF was substantially higher (HR 2.49; 95% CI: 1.83–3.38) compared to those without CKD (HR 1.38; 95% CI: 1.25–1.52), with a significant interaction (*p* for interaction = 0.0003). Subgroup-specific associations between gout and AF, along with interaction *p*-values, are visually summarized in Figure 2, and the corresponding hazard ratios and confidence intervals are detailed in Supplementary Table S1. These results underscore the synergistic effect of impaired renal function and systemic inflammation on atrial electrophysiological remodeling.

To assess the robustness of our findings, we conducted sensitivity analyses excluding individuals with <2 years of follow-up (*n* = 147,204), to account for potential early detection bias. The association between gout and AF remained consistent in this restricted cohort (adjusted HR 1.427; 95% CI: 1.299–1.570), suggesting that reverse causation was unlikely to account for the observed relationship.

## Discussion

Our study demonstrates a robust and independent association between gout and AF in a nationwide cohort of young Korean adults. This relationship persisted after adjusting for a wide range of potential confounding factors, including lifestyle variables and cardiometabolic comorbidities. The association was particularly pronounced in individuals without obesity and in those with chronic kidney disease (CKD), suggesting that gout-related arrhythmic risk may be modulated by systemic inflammation and renal function status (15, 16). These findings build on prior evidence and imply that, even among young and ostensibly healthy individuals, gout may represent a clinically relevant risk marker for early-onset atrial fibrillation.

Several prior studies in general adult populations have reported a positive association between gout and AF. For instance, a large U.S.-based cohort study by Singh and Cleveland (8) found that gout was associated with a modestly increased risk of AF among older adults (8). Similarly, studies utilizing Medicare claims data or electronic health records have supported the notion that hyperuricemia and inflammatory arthritis are linked to atrial arrhythmias (9, 16, 17). However, these studies primarily involved middle-aged or elderly cohorts, where AF incidence is already high and multiple overlapping risk factors complicate causal inference. In contrast, our study uniquely focuses on adults aged 20 to 39 years—a population in which AF is relatively rare. This age restriction minimizes confounding by age-related cardiovascular degeneration and provides further epidemiological data regarding the clinical association between these two conditions.

Although overt structural heart disease is uncommon before middle age, the minority of young adults who do develop AF face a disproportionately long exposure to stroke and heart-failure risk. Our findings suggest that gout may act as a plausible catalyst for AF development, as chronic hyperuricemia and low-grade inflammation can activate the NLRP3 inflammasome, increase oxidative stress, and promote atrial fibrosis and conduction heterogeneity, thereby lowering the arrhythmic threshold in structurally normal atria (18–21). These persistent, flare-independent processes offer a biologically coherent explanation for the excess AF risk seen in our cohort and underscore the importance of considering inflammatory-metabolic triggers when AF arises early in life. While these inflammatory and metabolic pathways are biologically plausible, our epidemiological data do not allow for a definitive mechanistic conclusion. Therefore, these potential mechanisms should be interpreted with caution.

The subgroup findings from our study provide further mechanistic insights. The stronger association observed in individuals without obesity may indicate that inflammation, rather than metabolic stress as an isolated factor, plays a more prominent role in the arrhythmogenic pathway of gout. Obesity is a known risk factor for AF, primarily through hemodynamic and structural changes such as atrial enlargement and epicardial fat deposition (22, 23). In obese individuals, the relative contribution of gout-related inflammation may be less apparent due to competing pathophysiologic factors (24). In contrast, among lean individuals, the pro-inflammatory effects of gout may exert a more direct influence on atrial electrophysiology.

Our results also highlight the substantial interaction between gout and CKD in relation to AF risk. CKD is a potent risk factor for AF through mechanisms including volume overload, sympathetic overactivity, uremic toxin exposure, and atrial structural remodeling (25, 26). Moreover, renal dysfunction exacerbates hyperuricemia by impairing uric acid excretion, creating a feedback loop that may amplify systemic inflammation and oxidative stress (27, 28). The synergistic effect observed in individuals with both gout and CKD underscores the importance of comprehensive risk factor management in this subgroup.

Hypertension, obesity, and CKD are well-established AF risk factors, and their co-occurrence with gout is not incidental. In individuals with gout, hypertension is frequently present and may contribute to atrial pressure overload, promoting left atrial enlargement and fibrotic change (29, 30). Obesity, as noted above, alters cardiac geometry and augments systemic inflammation. CKD, in turn, increases cardiac afterload and triggers neurohormonal activation. All three conditions are also associated with elevated serum uric acid levels, suggesting shared biological pathways. These interrelated mechanisms imply that in gout patients, aggressive management of blood pressure, weight, and renal function may not only improve joint outcomes but also reduce long-term arrhythmic risk (31).

This study has several notable strengths. First, it is one of the largest population-based investigations to date assessing the risk of AF in young adults with gout. The use of a comprehensive nationwide database allows for virtually complete follow-up and minimizes selection bias. The long follow-up duration strengthens the temporal relationship between exposure and outcome. We also adjusted for a broad range of clinical and lifestyle factors, reducing the likelihood of residual confounding. Importantly, our subgroup and sensitivity analyses showed consistent findings, enhancing the robustness of the results.

Nevertheless, some limitations should be acknowledged. First, gout and AF were identified from claims data, which may cause misclassification. However, strict diagnostic criteria improve specificity and have been validated in prior NHIS studies. Second, we lacked information on serum uric acid, flare frequency, medication use (e.g., colchicine, allopurinol), and echocardiographic findings (32). Therefore, we were unable to evaluate whether gout treatment modified the risk of incident AF. Third, residual confounding by unmeasured factors (e.g., genetics, diet) cannot be excluded (33, 34). Consequently, the combination of residual confounding, surveillance bias, lack of serum uric acid and medication data, and reliance on claims-based definitions prevents a firm conclusion that gout independently contributes to incident AF, and causal or mechanistic interpretations must be substantially tempered.

## Conclusion

In this large nationwide cohort of young Korean adults, gout was associated with an increased risk of atrial fibrillation (AF) after extensive adjustment. However, given the inherent limitations of claims-based data, these findings should be interpreted as a significant clinical association rather than a definitive causal link. While the association was prominent in those without obesity and with CKD, any mechanistic interpretations regarding arrhythmic vulnerability beyond traditional metabolic risk remain speculative and require further investigation. These findings highlight that gout may serve as a clinically relevant marker of increased cardiovascular and arrhythmic risk in young adults. Further studies are warranted to determine whether evaluating cardiovascular risk may improve outcomes in this population.

## Data Availability

The data analyzed in this study is subject to the following licenses/restrictions: The datasets used in this study are not publicly available due to data protection and privacy regulations. Access to the Korean National Health Insurance Service (NHIS) data is restricted and can be obtained only upon reasonable request and approval from the NHIS. Requests to access these datasets should be directed to Requests to access the dataset can be made through the Korean National Health Insurance Service (NHIS). Further information is available at: https://nhiss.nhis.or.kr.
