# Supplementary material for: Increased risk of atrial fibrillation in young adults with gout: a nationwide cohort study
Source: Front Cardiovasc Med. 2026 Jul 7;13:1862887. doi: 10.3389/fcvm.2026.1862887 (PMC13385323; doi:10.3389/fcvm.2026.1862887)
Supplement: Supplementary file 1 [file Table1.docx]

Supplementary Table 1. Subgroup Analyses of the Association Between Gout and Atrial Fibrillation

|  |  |  |  |  |  |  |  |
| --- | --- | --- | --- | --- | --- | --- | --- |
|  |  | N | Event | Duration | IR, 1000 PY | aHR (95% C.I) | P for interaction |
| **Age** |  |  |  |  |  |  | 0.4705 |
| <30 | No | 2,734,054 | 9,455 | 33,340,259 | 0.28 | 1 (Ref.) |  |
|  | Yes | 7,457 | 56 | 89,498 | 0.63 | 1.575 (1.211, 2.049) |  |
| >=30 | No | 3,736,925 | 25,772 | 46,212,193 | 0.56 | 1 (Ref.) |  |
|  | Yes | 28,285 | 390 | 345,497 | 1.13 | 1.421 (1.285, 1.571) |  |
| **Sex** |  |  |  |  |  |  | 0.1841 |
| Male | No | 3,836,768 | 26,483 | 47,356,521 | 0.56 | 1 (Ref.) |  |
|  | Yes | 31,751 | 418 | 386,977 | 1.08 | 1.417 (1.286, 1.562) |  |
| Female | No | 2,634,211 | 8,744 | 32,195,931 | 0.27 | 1 (Ref.) |  |
|  | Yes | 3,991 | 28 | 48,018 | 0.58 | 1.838 (1.268, 2.664) |  |
| **Smoking** |  |  |  |  |  |  | 0.4529 |
| No | No | 3,555,171 | 14,518 | 43,577,835 | 0.33 | 1 (Ref.) |  |
|  | Yes | 12,240 | 128 | 148,811 | 0.86 | 1.555 (1.306, 1.852) |  |
| EX | No | 666,750 | 5,242 | 8,277,898 | 0.63 | 1 (Ref.) |  |
|  | Yes | 6,141 | 88 | 75,516 | 1.17 | 1.306 (1.057, 1.612) |  |
| Current | No | 2,249,058 | 15,467 | 27,696,720 | 0.56 | 1 (Ref.) |  |
|  | Yes | 17,361 | 230 | 210,669 | 1.09 | 1.434 (1.259, 1.634) |  |
| **Alcohol consumption** |  |  |  |  |  |  | 0.0719 |
| Non | No | 2,444,109 | 11,505 | 30,018,356 | 0.38 | 1 (Ref.) |  |
|  | Yes | 10,923 | 142 | 132,354 | 1.07 | 1.676 (1.420, 1.979) |  |
| Mild | No | 3,459,058 | 19,067 | 42,551,426 | 0.45 | 1 (Ref.) |  |
|  | Yes | 19,612 | 231 | 239,473 | 0.96 | 1.391 (1.221, 1.584) |  |
| Heavy | No | 567,812 | 4,655 | 6,982,671 | 0.67 | 1 (Ref.) |  |
|  | Yes | 5,207 | 73 | 63,169 | 1.16 | 1.231 (0.977, 1.552) |  |
| **Regular Exercise** |  |  |  |  |  |  | 0.9199 |
| No | No | 5,641,318 | 29,964 | 69,326,781 | 0.43 | 1 (Ref.) |  |
|  | Yes | 29,946 | 363 | 364,021 | 1.00 | 1.435 (1.293, 1.592) |  |
| Yes | No | 829,661 | 5,263 | 10,225,671 | 0.51 | 1 (Ref.) |  |
|  | Yes | 5,796 | 83 | 70,974 | 1.17 | 1.453 (1.169, 1.805) |  |
| **Obesity** |  |  |  |  |  |  | **0.0007** |
| No | No | 4,771,339 | 21,188 | 58,671,024 | 0.36 | 1 (Ref.) |  |
|  | Yes | 15,421 | 169 | 188,086 | 0.90 | 1.754 (1.507, 2.041) |  |
| Yes | No | 1,699,640 | 14,039 | 20,881,428 | 0.67 | 1 (Ref.) |  |
|  | Yes | 20,321 | 277 | 246,909 | 1.12 | 1.258 (1.116, 1.417) |  |
| **Abdominal Obesity** |  |  |  |  |  |  | 0.1349 |
| No | No | 5,635,468 | 27,187 | 69,324,465 | 0.39 | 1 (Ref.) |  |
|  | Yes | 23,099 | 241 | 282,113 | 0.85 | 1.489 (1.311, 1.691) |  |
| Yes | No | 835,511 | 8,040 | 10,227,987 | 0.79 | 1 (Ref.) |  |
|  | Yes | 12,643 | 205 | 152,882 | 1.34 | 1.290 (1.123, 1.482) |  |
| **Diabetes mellitus** |  |  |  |  |  |  | 0.1502 |
| No | No | 6,347,435 | 33,775 | 78,039,851 | 0.43 | 1 (Ref.) |  |
|  | Yes | 33,775 | 411 | 411,363 | 1.00 | 1.471 (1.334, 1.622) |  |
| Yes | No | 123,544 | 1,452 | 1,512,601 | 0.96 | 1 (Ref.) |  |
|  | Yes | 1,967 | 35 | 23,632 | 1.48 | 1.138 (0.814, 1.592) |  |
| **Hypertension** |  |  |  |  |  |  | 0.139 |
| No | No | 5,993,771 | 29,533 | 73,685,886 | 0.40 | 1 (Ref.) |  |
|  | Yes | 26,912 | 279 | 328,184 | 0.85 | 1.522 (1.353, 1.713) |  |
| Yes | No | 477,208 | 5,694 | 5,866,567 | 0.97 | 1 (Ref.) |  |
|  | Yes | 8,830 | 167 | 106,811 | 1.56 | 1.315 (1.127, 1.534) |  |
| **Dyslipidemia** |  |  |  |  |  |  | 0.3999 |
| No | No | 6,034,322 | 31,491 | 74,169,848 | 0.42 | 1 (Ref.) |  |
|  | Yes | 28,372 | 328 | 345,605 | 0.95 | 1.474 (1.322, 1.644) |  |
| Yes | No | 436,657 | 3,736 | 5,382,605 | 0.69 | 1 (Ref.) |  |
|  | Yes | 7,370 | 118 | 89,390 | 1.32 | 1.345 (1.120, 1.616) |  |
| **CKD** |  |  |  |  |  |  | **0.0003** |
| No | No | 6,322,942 | 34,250 | 77,639,080 | 0.44 | 1 (Ref.) |  |
|  | Yes | 34,189 | 403 | 415,876 | 0.97 | 1.378 (1.248, 1.520) |  |
| Yes | No | 148,037 | 977 | 1,913,372 | 0.51 | 1 (Ref.) |  |
|  | Yes | 1,553 | 43 | 19,119 | 2.25 | 2.490 (1.834, 3.382) |  |
|  |  |  |  |  |  |  |  |

Note: Values represent adjusted hazard ratios (aHRs) and incidence rates (IRs) per 1,000 person-years (PY) across subgroups stratified by demographic, behavioral, and clinical factors. All estimates are derived from fully adjusted Cox proportional hazards models (Model 3), which include adjustments for age, sex, BMI, income level, smoking status, alcohol consumption, regular exercise, diabetes mellitus, hypertension, dyslipidemia, and chronic kidney disease (CKD).

P-values for interaction test for effect modification between subgroup characteristics and gout status.

Abbreviations: IR = incidence rate; PY = person-years; aHR = adjusted hazard ratio; CKD = chronic kidney disease.
