# Supplementary material for: Increased risk of atrial fibrillation in young adults with gout: a nationwide cohort study
Source: Front Cardiovasc Med. 2026 Jul 7;13:1862887. doi: 10.3389/fcvm.2026.1862887 (PMC13385323; doi:10.3389/fcvm.2026.1862887)
Supplement: Supplementary file 2 [file Supplementaryfile1.docx]

Supplementary Figure 1. Flowchart of the Study Population Selection

Korean adults aged 20–39 years who underwent health screening (2009–2012)

(n = 6,891,401)

Excluded: Missing screening variables

(n = 328,447)

Excluded: Diagnosed with AF within 1 year after index date (1-year lag)

(n = 4,124)

Remaining after exclusion

(n = 6,562,954)

Final study population
(n = 6,506,721)

Gout group
(n = 35,742)

Non-gout group
(n = 6,470,979)

Excluded: Non-gout participants with a single claim of gout diagnosis

(n = 28,342)

Excluded: History of atrial fibrillation before index date (wash-out)

(n = 23,767)

Remaining after AF wash-out

(n = 6,539,187)

Remaining after 1-year lag period

(n = 6,535,063)
